# Midodrine as adjunctive support for treatment of refractory hypotension in the intensive care unit: a multicenter, randomized, placebo controlled trial (the MIDAS trial)

**DOI:** 10.1186/s12871-017-0339-x

**Published:** 2017-03-21

**Authors:** Matthew H. Anstey, Bradley Wibrow, Tharusan Thevathasan, Brigit Roberts, Khushi Chhangani, Pauline Yeung Ng, Alexander Levine, Alan DiBiasio, Todd Sarge, Matthias Eikermann

**Affiliations:** 1 Sir Charles Gairdner Hospital, Perth, WA Australia; 2 School of Medicine and Pharmacology, University of Western Australia, Perth, Australia; 3 Department of Anesthesia, Critical Care and Pain Medicine, Massachusetts General Hospital and Harvard Medical School, Boston, USA; 4 Department of Pharmacy Practice and Administration, University of Saint Joseph, Hartford, CT USA; 5 Department of Pharmacy, Massachusetts General Hospital, Boston, USA; 6 Department of Anesthesia and Critical Care, Beth Israel Deaconess Medical Center, Boston, USA; 7 Universitaet Duisburg Essen, Klinik fuer Anaesthesiologie und Intensivmedizin, Essen, Germany

**Keywords:** Midodrine, Alpha adrenergic agonist, Vasopressor, Hypotension, Intensive care unit, High dependency unit

## Abstract

**Background:**

Patients admitted to intensive care units (ICU) are often treated with intravenous (IV) vasopressors. Persistent hypotension and dependence on IV vasopressors in otherwise resuscitated patients lead to delay in discharge from ICU. Midodrine is an oral alpha-1 adrenergic agonist approved for treatment of symptomatic orthostatic hypotension. This trial aims to evaluate whether oral administration of midodrine is an effective adjunct to standard therapy to reduce the duration of IV vasopressor treatment, and allow earlier discharge from ICU and hospital.

**Methods:**

The MIDAS trial is an international, multicenter, randomized, double-blind, placebo-controlled clinical trial being conducted in the USA and Australia. We are targeting 120 patients. Adult patients admitted to the ICU who are resuscitated and otherwise stable on low dose IV vasopressors for at least 24 h will be considered for recruitment. Participants will be randomized to receive midodrine (20 mg) or placebo three times a day, in addition to standard care. The primary outcome is time (hours) from initiation of midodrine or placebo to discontinuation of IV vasopressors. Secondary outcomes include time (hours) from ICU admission to discharge readiness, ICU length of stay (LOS) (days), hospital LOS (days), rates of ICU readmission, and rates of adverse events related to midodrine administration.

**Discussion:**

Midodrine is approved by the Food and Drug Administration (FDA) for the treatment of symptomatic orthostatic hypotension. In August 2010, FDA proposed to withdraw approval of midodrine because of lack of studies that verify the clinical benefit of the drug. We obtained Investigational New Drug (IND 113,330) approval to study its effects in critically ill patients who require IV vasopressors but are otherwise ready for discharge from the ICU. A pilot observational study in a cohort of surgical ICU patients showed that the rate of decline in vasopressor requirements increased after initiation of midodrine treatment. We hypothesize that midodrine administration is effective to wean IV vasopressors and shorten ICU and hospital LOS. This trial may have significant implications on lowering costs of hospital care and obtaining FDA approval for new indications for midodrine.

**Trial Registration:**

This study has been registered at clinicaltrials.gov on 02/09/2012 (NCT01531959).

## Background

Treatment with vasopressors is one of the most common reasons for admission to an intensive care unit (ICU) [1]. Persistent hypotension in otherwise resuscitated patients can be a delay to discharging patients from the ICU. The addition of oral midodrine may be a solution to reduce the duration of time the patient remains on IV vasopressors and ICU length of stay (LOS).

Midodrine, an oral alpha-1 adrenergic agonist, has approval by the United States Federal Drug Administration (FDA) for the treatmentof symptomatic orthostatic hypotension [2]. Midodrine is a prodrug that is metabolized to form desglymidodrine, with peak blood levels of midodrine reached within 30 min and the active metabolite peaking about 1 to 2 h after administration [3]. It has a relatively safe side effect profile with minimal central nervous system side effects, and good oral bioavailability [4].

Midodrine has previously been evaluated in a series of prospective trials for orthostatic hypotension. Jankovic et al. found a dose of 10 mg three times daily (TDS) increased standing systolic blood pressure by 22 mmHg versus placebo [5]. Wright et al. conducted a dose response study and found that midodrine (10 mg and 20 mg) increased standing blood pressure compared with placebo [3].

Midodrine has also been studied in the setting of dialysis-induced hypotension, and administration of 2.5-10 mg prior to dialysis improved the symptoms of intradialytic hypotension and increased post-dialysis systolic blood pressure [6].

In the critical care environment, the authors have obtained an Investigational New Drug (IND) approval from the United States FDA (IND 113,330; date of receipt: September 12, 2011) to study its effects in patients who require low dose IV vasopressors following resuscitation. We then conducted an observational study on the use of oral midodrine to hasten weaning of IV vasopressors in a cohort of surgical ICU patients [7]. In this prospective observational study, 20 patients who met surgical ICU discharge criteria except for persistent requirement for IV vasopressors (phenylephrine <150 mcg/min or noradrenaline <8 mcg/min) received oral midodrine (modal dose 20 mg, range 5–20 mg) TDS. Requirements for IV vasopressors were assessed following each of the first four doses of midodrine. The rates of decline of vasopressor requirement in the 12 h preceding midodrine, and from initiation of midodrine until 4 h post the fourth dose were calculated. 6 of 20 study patients were able to be weaned from vasopressors following the first dose of midodrine, increasing to 14 of 20 following the fourth dose. The rate of decline in vasopressor requirements increased from 0.62 mcg/min per hour to 2.20 mcg/min per hour (*p* = 0.012), meaning less IV vasopressor was required. Mean arterial pressure (MAP), heart rate (HR), and total body fluid balance were not significantly different pre and post midodrine administration, suggesting that changes in hemodynamic status or fluid resuscitation were not responsible for the change in the rate of decline in vasopressor requirements.

Cardenas et al. reported on the use of midodrine to wean requirement of IV vasopressors in a medical ICU population [8]. During a six month period, 50 patients who met discharge criteria except for the use of low to moderate dose of a single IV vasopressor were administered oral midodrine (average initial and maximal doses 30.5 mg and 66.6 mg per day). Midodrine was continued on the ward and reduced as tolerated by the admitting team. No adverse effects were observed. In this study, 27 (54%) of the patients were discharged from the hospital on midodrine (average dose 17.8 mg per day). Another recent retrospective study from a medical ICU included 275 patients over one year, with a diagnosis of septic shock and required at least 24 h of IV vasopressors, who were stable or on reducing doses of IV vasopressors. The authors compared patients on IV vasopressors only to those who received IV vasopressors plus adjunctive midodrine (with a starting dose of 10 mg TDS) and showed a reduction in the mean IV vasopressor duration (2.9 days versus 3.8 days, *p* < 0.001) and reduced ICU length of stay (7.5 versus 9.4 days, *p* = 0.017) in the IV vasopressor plus midodrine group [9].

There are also a case report and two case series of the use of midodrine to wean from or prevent the need for IV vasopressors. O’Donnell reported on the use of midodrine 10 mg TDS to wean a patient from noradrenaline infusion post C7 to T6 laminectomy for spinal cord compression secondary to a leukemic deposit [10]. Sharma reported the use of oral midodrine 10 mg TDS to wean IV dopamine infusions in three patients with hypotension secondary to myocardial stunning post percutaneous coronary intervention [11]. Sharma also reported on the use of oral midodrine for hypotension post carotid artery stenting. Four patients with persistent hypotension post carotid artery stenting were successfully treated with midodrine 10 mg TDS, avoiding the requirement for ICU admission (the preceding 11 cases required IV dopamine infusions in ICU) [12].

The primary objective of this trial is to determine whether addition of midodrine to standard care reduces the time to discontinuation of IV vasopressor use. We will also study whether hospital and ICU LOS are decreased, as well as rates of ICU or high dependency unit (HDU) readmission, and rates of adverse events related to the use of midodrine (such as bradycardia and other hemodynamically significant arrhythmias).

## Methods

### Study design and setting

**Fig. 1:**
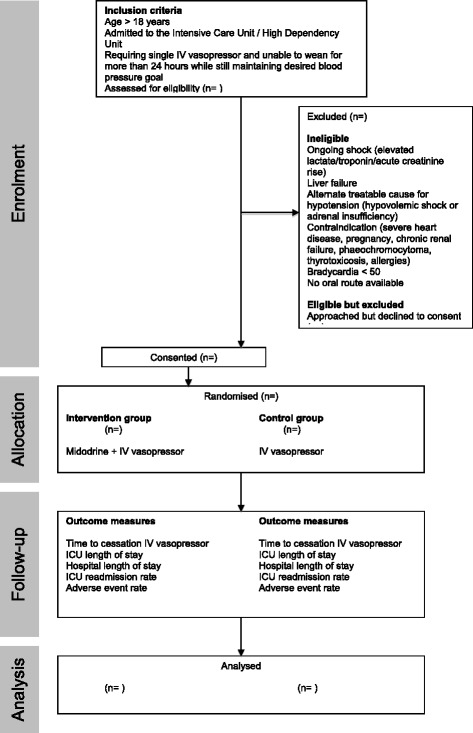
Outline of study design

This study will be an international, multicenter, randomized, double-blind, placebo-controlled intervention trial. An outline of the study design is provided in Fig. 1. Study recruitment commenced in October 2012 at Massachusetts General Hospital in Boston, United States of America. We started enrolling patients at Sir Charles Gairdner Hospital, Western Australia in 2016. There is expectation of expansion to another academic medical center. The trial is expected to be completed by June 2017.

The IND application for midodrine (IND 113,330) was approved by the United States FDA on Sep 12, 2011. A notification to conduct the trial under the Clinical Trials Notification (CTN) Scheme (Application ID CT-2016-CTN-00226-1 v3) was acknowledged by the Australian Government Therapeutic Goods Administration (TGA). Ethics approval has been sought and obtained from the ethics committees of participating hospitals (Partners IRB Protocol #2011P002049, and Sir Charles Gairdner Hospital HREC Protocol #2015-098).

### Study population

A local investigator will screen ICU patients daily. Adult patients admitted to the ICU or HDU who are hypotensive and requiring IV vasopressors will be considered for the study. A total of 120 patients will be recruited.

### Inclusion and exclusion criteria

All adult patients who are hypotensive (systolic blood pressure <90 mmHg) and require IV vasopressors for more than 24 h are eligible for study inclusion. They should be otherwise resuscitated and have stable blood pressure on single agent infusion of noradrenaline <8 mcg/min, or phenylephrine <100 mcg/min, or metaraminol <60 mcg/min). Table 1 outlines the exclusion criteria.

**Table 1:** Study exclusion criteria

Exclusion criteria	Justification
Clinical evidence of inadequate tissue oxygenation	For safety consideration, patients with ongoing shock excluded.
Hypovolemic shock or hypotension due to adrenal insufficiency (based on clinical suspicion or available routine testing)	Treatable cause of hypotension.
Clinical evidence of liver failure	Midodrine metabolism occurs predominantly in liver.
Pregnancy	No safety data available.
Chronic renal failure (serum creatinine >2 mg/dL, or 180umol/L)	Midodrine and its active metabolite are almost completely excreted in urine. Drug accumulation may occur, and for this trial dose adjustment is not possible.
Severe organic heart disease (ejection fraction less than 30%)	Contraindicated due to higher risk of arrhythmias
Acute urinary retention	Accumulation of drug may occur as usually almost completed excreted in urine.
Pheochromocytoma (based on clinical suspicion)	Contraindication.
Thyrotoxicosis (based on clinical suspicion)	Contraindication.
Co-enrolment in another vasopressor clinical trial	Confounding factor.
Midodrine as pre-admission medication	Confounding factor.
Any known allergies to midodrine	Contraindication.
Bradycardia (HR <50/min)	Higher likelihood of symptomatic bradycardia.
No enteral route available	Oral preparation.

### Intervention

**Fig. 2:**
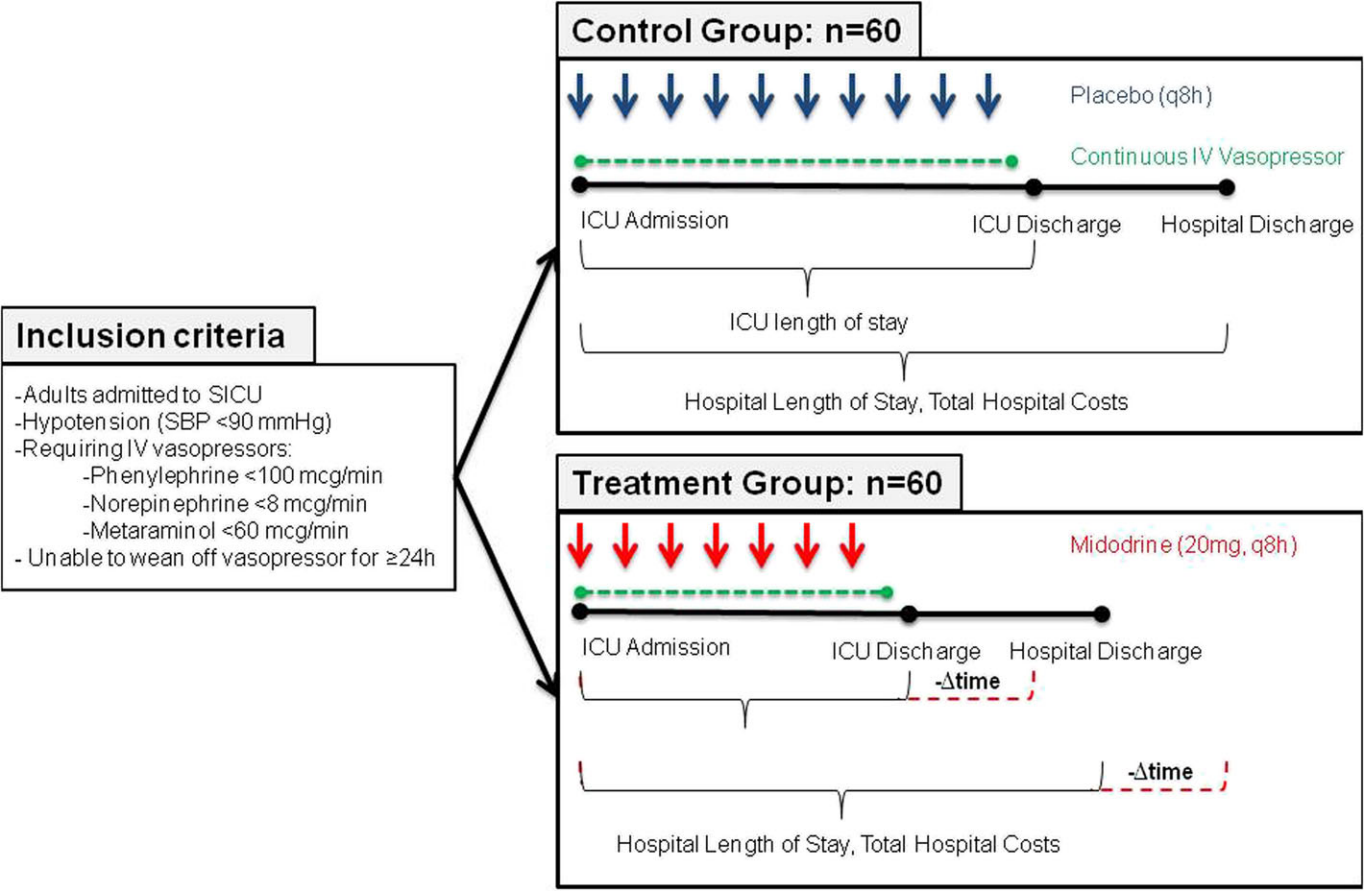
Flowchart of study procedures

A target blood pressure (systolic blood pressure (SBP) or MAP) is determined by the treating physician at enrollment of each patient. Eligible participants will be randomized to receive midodrine 20 mg daily or placebo three times daily, in addition to continuation of the IV vasopressor, until the primary outcome (cessation of IV vasopressors) or an adverse event occurs. The study drug and placebo will be delivered in the form of non-identifying capsules, containing either the active ingredient midodrine, or lactose powder. All patients will receive usual supportive care (intravenous fluids, antibiotics etc.) as per the treating physicians and standard practice. A flowchart of the study procedures is shown in Fig. 2.

The study drug will be delivered in a double-blind manner until:

1. Primary outcome is reached – cessation of IV vasopressors; or
2. Patient is discharged from the ICU; or
3. Patient develops further hypotension – requiring noradrenaline >8 mcg/min, phenylephrine >100 mcg/min, or metaraminol >60 mcg/min; or
4. Adverse event occurs – subject develops signs and symptoms of organ failure or hypoperfusion, adverse events related to midodrine including serious allergic reaction, or death.

### Blinding and randomization

The principal investigator or research coordinator at each participating site will enroll patients. All study personnel will be blinded, except the investigational pharmacist, to the treatment group of the subjects. Patients will be randomly assigned in a 1:1 ratio to receive either midodrine or placebo. Randomization will be performed with the use of a computer-generated randomization list and will be stratified by site. An independent statistician not responsible for analyzing the data will create the allocation sequence. This list will be provided by the independent statistician directly to the compounding pharmacy. Individual patient’s drug bottles will be labeled with site-specific randomization numbers, which will become the patient’s study number. Study sites will dispense the study drug bottles sequentially to patients in the order that they are included in the study.

### Outcomes

The primary outcome is time (hours) from initiation of midodrine or placebo to discontinuation of IV vasopressors. Secondary outcomes include time (hours) from ICU admission to discharge readiness, ICU LOS (days), hospital LOS (days), rates of readmission to ICU or HDU, and rates of adverse events related to midodrine administration. A cost consequence analysis will also be performed by a medical economist based on the ICU and hospital LOS.

### Statistical analysis

The sample size estimation is informed by the previous observational study by Levine et al. [7]. In this study, the rates of vasopressor administration decreased by 38% from −0.62 ± 1.40 mcg/min per hour to −2.20 ± 2.45 mcg/min per hour after addition of midodrine. Based on a median time of 17 h from midodrine initiation to discontinuation of IV vasopressors, the 38% reduction translates to an estimated 6 h difference. 6 h is also thought to be a meaningful time frame in terms of reduction in duration of vasopressor treatment. Accordingly, we expect that a sample size of 50 subjects per group will provide us with >80% probability (power) to detect a difference of 6 h in the primary outcome. Allowing for drop-out, our target sample size is 120.

The study analysis will be by intention to treat. We will analyze our data by using a Student’s *t*-test to determine whether time from initiation of midodrine until discontinuation of IV vasopressors, time from ICU/HDU admission to ICU/HDU discharge readiness, and ICU and hospital LOS differs between groups. Using a Chi-Square test, we will test whether rates of ICU/HDU readmission and incidence of adverse events is different between treatment groups. Time-dependent survival analysis will be employed to assess relationships between addition of midodrine/placebo and ICU/hospital LOS, with mortality considered as the time-dependent covariate. A P value of <0.05 will be considered statistically significant. All analyses will be conducted by a statistician blinded to intervention group assignment.

### Data collection

Baseline descriptive data collection will occur on day of enrolment and includes age, gender, ethnicity, APACHE II score, admission diagnosis, pre-existing co-morbidities (diabetes, coronary artery disease, asthma, peripheral vascular disease, renal failure, psychiatric disease, musculoskeletal disease, and others) obtained from the subject, the subject’s family, and the subject’s medical chart. Baseline laboratory data will be recorded (hemoglobin, white cell count, alanine aminotransferase (ALT), international normalized ratio (INR), bilirubin, urea, creatinine, troponin, lactate). While in the study, hourly blood pressure, heart rate, dose of intravenous vasopressor and daily SOFA score will be recorded. The main outcome measure, time in hours from initiation of midodrine until discontinuation of IV vasopressors, will be recorded. To assess for potential confounders, we will also record presence, and time of removal, of epidural anesthesia, and any blood transfusion. At the time of ICU discharge, the following variables will be collected: time of ICU discharge readiness and ICU LOS. After discharge, follow-up variables will be rates of ICU readmission and hospital LOS and any other complications. Data will be uploaded to a secure database (REDCap) [13]. Data stored on REDCap are coded without protected information. The center at Massachusetts General Hospital owns the REDCap database, and is responsible for all data entry and monitoring.

### Safety

The patient will be kept in the ICU for observation for 24 h following cessation of study drug to reduce the likelihood of hypotension or other side effects on discharge. If the blood pressure goal is met for more than 24 h without IV vasopressors, the study drug will be discontinued prior to discharge to the ward. The accepting team will be informed on discharge that the patient had received a study drug. Instructions will be given to medical and nursing staff to contact a physician investigator if the subject becomes hypotensive in the 24 h after ICU discharge (defined as a SBP <90 mmHg).

### Adverse events

Adverse events will be recorded daily. Nursing charts and documentation, as well as physicians’ notes will be reviewed daily to identify potential adverse events. Adverse events are defined as any unfavorable and unintended signs, symptoms or diseases chronologically associated with midodrine or placebo administration. The relationship of any adverse event to the study drug will be assessed by the investigator and qualified by strength of association (not related, unlikely related, possibly related, probably related, or definitely related) and severity of the event (mild, moderate or severe). All adverse events will be summarized by the study staff and, where appropriate, reported to the local human research committee. Serious adverse events (SAE) are hypertension (increase in SBP 20% higher than the predefined goal set by the primary team, or >160 mmHg), bradycardia (decrease in heart rate 20% lower than the predefined goal set by the primary team or <40 beats per minute), hemodynamically significant tachyarrythmias (resulting in drop in SBP >20 mmHg), or new organ failure (evidence of inadequate organ oxygenation, liver and renal failure as per definitions in exclusion criteria).

### Data and safety monitoring board

Data and safety monitoring board (DSMB) has been created and comprises of physicians with relevant critical care experience. The members are not involved in the study and will be completely independent of any study-related patient recruitment and data collection.

## Ethics

### Consent

As this is an international multicenter trial, the process of informed consent may be different between sites, and will be dependent on national and cultural standards. Informed consent will be obtained from the patient, or when the patient is unable to consent, from the patient’s proxy. A copy of the IRB approved informed consent form will be given to the patient and/or healthcare proxy. All patients will be followed prospectively and given the opportunity to consent to continue in the study following resolution of their critical illness and capacity is regained.

## Discussion

This article presents the protocol and data analysis plan for the MIDAS trial; an international, multicenter, randomized, double-blind, placebo-controlled clinical trial evaluating the addition of midodrine to IV vasopressor treatment in otherwise stable ICU patients.

Midodrine, an oral alpha-1 adrenergic agonist, was approved for the treatment of symptomatic orthostatic hypotension under the United States FDA’s accelerated approval regulations in 1996 [2]. In August 2010, the FDA proposed to withdraw approval because of the lack of post-approval studies that confirm the clinical benefit of the drug [14]. It was the first time the agency had issued such a notice. Subsequent to support for its use from patients, doctors and professional organizations, the FDA reversed its decision to withdraw approval [15], but this decision was met with mixed response [16, 17].

Over recent years, there has been increasing interest in using midodrine to help patients wean off or avoid IV vasopressors [6, 10–12]. The drug has a reasonable safety profile, its main side effects being pilomotor reaction, urinary retention, and supine hypertension. The use of an oral vasopressor such as midodrine in addition to IV vasopressors in otherwise resuscitated and stable ICU patients has several reasons for attractiveness. Firstly, it helps to avoid some of the potential complications of administering IV vasopressors, such as risks of central line insertion and catheter-related bloodstream infections. Secondly, they may help to shorten the duration of IV vasopressor treatment, and hence reduce ICU length of stay and ICU-acquired complications. This has significant cost implications for the patient and hospital.

We communicated directly with the United States FDA and obtained IND approval to study the use of midodrine in the ICU setting. In our pilot observational study [7], we detected that midodrine treatment was associated with an increase in the magnitude of decline of the IV vasopressor rate. This pilot study offered promising data, but was limited in its small sample size (20 patients) and lack of a control group. We designed the MIDAS trial as a pragmatic clinical trial to study its use in routine patient care conditions. We have designed the inclusion and exclusion criteria so they can be applied to a broad clinical setting across a wide range of patients. We hope to improve the generalizability of our results by carrying out the study in a multicentric design.

One limitation of this study is the fixed drug dosing. This was selected to simplify the protocol and it was shown to be safe during the pilot study. However, an individualized dose titrated according to renal or hepatic function may allow midodrine to be used in a wider range of patients. A second limitation is we do not have a standardized protocol with regards to blood pressure targets for initiation, escalation, or weaning of IV vasopressors. The different blood pressure goals set by the primary care team may confound the individual duration of vasopressor treatment. A third limitation is that we are not studying the use of this drug on the ward environment, which is another useful indication of midodrine in preventing ICU admission.

## Conclusion

This manuscript provides a description of our study protocol and analysis plans for the MIDAS international, multicenter, randomized placebo- controlled clinical trial. This trial is an opportunity to improve the efficiency of our ICU beds and healthcare costs by facilitating early and safe cessation of intravenous vasopressors in ICU patients.

## Abbreviations

DSMB: Data and safety monitoring board
FDA: Food and Drug Administration
HDU: High dependency unit
HR: Heart rate
ICU: Intensive care unit
IV: Intravenous
LOS: Length of stay
MAP: Mean arterial pressure
SBP: Systolic blood pressure
TDS: Three times daily
